# The influence of foot muscles exercises and minimalist shoes on lactate threshold velocity in long-distance amateur runners: a randomized controlled trial

**DOI:** 10.1038/s41598-024-67525-5

**Published:** 2024-07-17

**Authors:** Iwona Sulowska-Daszyk, Bartosz Zając, Anna Mika

**Affiliations:** 1 Institute of Clinical Rehabilitation, University of Physical Education, Al. Jana Pawla II 78, 31-571 Krakow, Poland; 2 Laboratory of Functional Diagnostics, Central Scientific and Research Laboratory, University of Physical Education, 31-571 Kraków, Poland

**Keywords:** Public health, Therapeutics

## Abstract

The exercises of plantar foot muscles may have beneficial effects on the performance of the lower extremity muscles. The aim of this study was to compare two methods of foot muscle strengthening: direct short foot muscle exercises and indirect activation through training in minimalist footwear in regard to influence on lactate threshold velocity in long-distance runners. 55 recreational runners aged 21–45 years took part in that study. They were randomly divided into 2 groups: Group 1 (n = 25) with short foot muscle exercises, and Group 2 (n = 30) with training in minimalist shoes. The progressive running test was performed to determine heart rate (HR) and running velocity corresponding to lactate threshold (VLT). Two-way ANOVA was used to determine the significance of the differences regarding the evaluated variables. After the 8-week training program, higher values of VLT were observed in both groups. This change was significant only in Group 1 (p < 0.05). In Group 2, the higher value was noted but the changes were non-significant. Strengthening of the short foot muscles may improve lactate threshold velocity which is connected with running performance. Considering the obtained results, it is worth contemplating the implementation of these methods in the training of long-distance runners.

## Introduction

Running economy is commonly defined as the steady-state oxygen uptake for a given velocity of submaximal running^1^. However, in some studies conducted among endurance athletes, such as runners or cyclists, it has been reported that velocity at lactate threshold (V LT), rather than VO2 peak, is more closely correlated to endurance performance. The VO2 maintained during an endurance run is equal to the product of the runner’s VO2 peak and the percentage of VO2 peak that can be maintained during the performance. The second parameter is related to the VO2 measured at the lactate threshold (LT), so that for endurance sports, the performance VO2 is closely linked to the VO2 at the LT^2^. Increased V LT theoretically indicates a higher average running intensity without accumulating lactate^3^ and may be better related to success in running. Scientists widely agree that an increase in the lactate threshold typically results in improved endurance performance^4–6^.

It has been reported that running barefoot or in minimal shoes is slightly less costly than running in standard cushioned shoes. It is suggested that runners who wear standard cushioned stiff-soled shoes with arch supports have weaker intrinsic foot muscles than runners who are habitually barefoot or minimally shod^7^. A similar relationship also occurs in non-runners who regularly use minimalist footwear in their daily activities^8^. Although the importance of intrinsic foot muscle strengthening has been discussed, there are very few studies in which the influence would be assessed regarding this type of intervention on running economy and performance^9–12^. Some authors indicated a positive effect of short foot muscle exercises on results in short- and long-run tests^10,13,14^.

There are few studies on the effect of running in minimalist footwear on the short foot muscles. The results showed an increase in muscle thickness and the cross-sectional area^15,16^ and increase in toe flexion strength^17,18^. Some authors have also emphasized that increased somatosensory feedback and external cushioning removement in minimalist footwear may enhance running economy and reduce the risk of injury^19^.

The majority of research to date has been mainly focused on the effect of foot-strike pattern or single use of different types of footwear on running economy and performance. Our study is one of the few in which the effect of long-term training in minimalist footwear has been evaluated. Moreover, there are only few studies on the effect of intrinsic foot muscle strengthening exercises on running economy as well as short- and long-run performance.

Therefore, the aim of this study was to compare two methods of foot muscle strengthening: direct short foot muscle exercises and indirect activation through training in minimalist footwear. We also wanted to evaluate whether two such interventions have an effect on running performance in terms of influence on lactate threshold and running velocity associated with lactate threshold in long-distance runners.

## Methods

**Figure 1:**
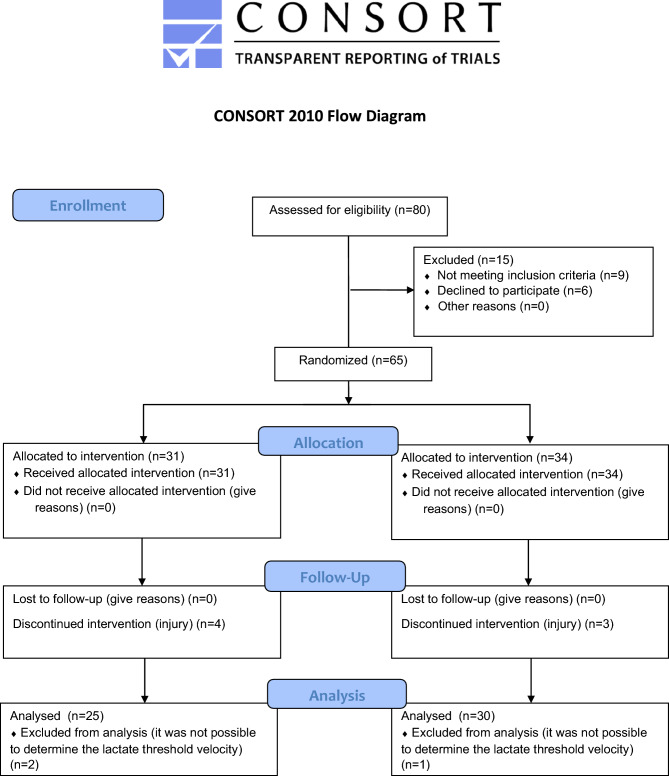
Consort diagram.

The study included 55 long-distance, recreational runners (18 females and 37 males), aged 21–45 years (mean ± SD 35.75 ± 6.26), who regularly run a total distance of 20–100 km (km) per week (mean ± SD 47.49 km ± 20.72 km) (Fig. 1).

The inclusion criteria were as follows: age between 20 and 45 years, regular running training (at least 3 running session per week, with a minimum training time of 30 min), a weekly running distance of 20 km or more and consent to participate in the study. The exclusion criteria were as follows: diagnosed foot deformation, using minimalist shoes or short foot muscle exercises before starting the project, previous history of injury in the last 6 months, systemic disease (e.g., fibromyalgia, hypertension, diabetes), chronic pain.

All participants were informed in detail about the research procedures and purpose of the study before providing their written informed consent. All the procedures complied with the 1964 Declaration of Helsinki. The study was registered in the Australian New Zealand Clinical Trials Registry (ACTRN12621001450808, date registered: 25/10/2021) and approval of the ethics committee of Regional Medical Chamber had been obtained (No. 70/KBL/OIL/2021).

The study participants were randomly divided into 2 groups: Group 1 (n = 25), in which participants performed short foot muscle exercises (SF Group), and Group 2 (n = 30), in which participants carried out running training in minimalist shoes (MS Group). The researchers used simple randomization by flipping a coin. The researcher who performed the measurements was blinded to the participant group allocation. A detailed characterization of both groups is presented in Table 1.

**Table 1:** Detailed characteristics of the groups.

	Group 1 (SF group) (n = 25) Mean ± SD	Group 2 (MS group) (n = 30) Mean ± SD	p
Age	35.40 ± 7.14	36.03 ± 5.53	0.982
Males	14	23	
Females	11	7	
Height (cm)	173.94 ± 8.75	177.57 ± 7.18	0.091
Body mass (kg)	72.15 ± 14.56	72.07 ± 10.25	0.635
Total distance covered per week (km)	45.52 ± 18.02	49.13 ± 22.90	0.671

*SD* standard deviation, *cm* centimeters, *kg* kilograms, *km* kilometers, *SF Group* group with short foot muscle exercises, *MS Group* group with running training in minimalist shoes, *p* p value for t-test for independent groups.

During the experiment, participants in both groups performed running training adjusting to their capabilities, at a frequency of 3–5 × per week. The study participants were not members of sports clubs but trained independently. Participants were asked to maintain a consistent intensity and volume of training throughout the duration of the experiment. They were also asked to create a profile on the Strava sports application (Strava, San Francisco, California), where the particular distance and level of intensity of the completed workouts were recorded. This was monitored by the researcher, who had access to the profile of each study participant.

In Group 1 (SF Group), all measurements were performed at baseline, after 8 and 16 weeks. For the first 8 weeks, participants continued their running training, which was monitored by researchers. After that, the second measurement was performed and then the short foot muscle exercises program, lasting 8 weeks, started^20,21^. All exercises were carried out barefoot. The exercise protocol was performed every day and lasted approximately 30 min. Every day, the participants received a reminder about doing exercises, which they confirmed in the diary. Moreover, once a week, the training was conducted by a physical therapist, who examined the correctness of exercises performance. Every two weeks there was a progression of exercises program by changing position, as well as increasing the load and the number of repetitions.

The exercise protocol was an extension of a previously proposed program^22^. It included activating the internal foot muscles through exercises without equipment (for example the Short Foot Exercise, the Vele’s Forward Lean)^20,21^ and exercises on a stability disc. In each exercise, participants paid attention to evenly load the heads of the 1^st^ and 5^th^ metatarsals. Before each training session, the participants performed plantar self-myofascial release of each foot for 3 min using a tennis ball. The selected exercises have been presented in Table 2.

**Table 2:**
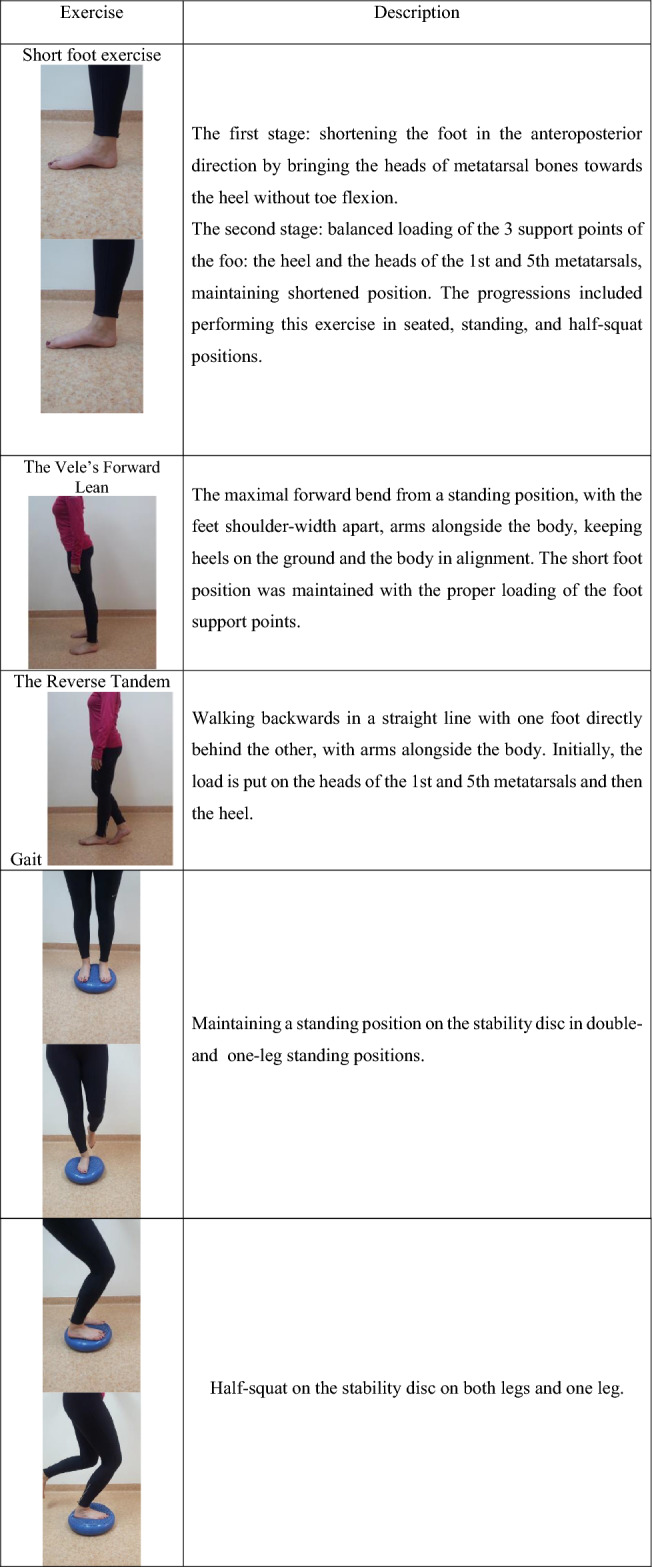
Examples of used exercises in the SF group.

The study participants from Group 2 (MS Group) were familiarized with the minimalist footwear walking and running protocol and received verbal and written instructions. All measurements were conducted 4 times: at baseline, after 8, 12 and 20 weeks. After the first measurement, the runners continued their running training routine which did not change throughout the duration of the experiment. A proper size of the Vibram Five Fingers (VFF) was selected for each participant. After the second measurement, the runners received their pair of shoes and there was a 4-week adaptation phase of walking in minimalist shoes, which included progression by increasing the time of walking in the VFF. The minimalist shoes were used every day, starting with 5 min per day and ending with 1 h of walking. Every day, the participants received a reminder about the requirement to use the minimalist shoes, which they confirmed in the diary. After the end of the stage, there was a third measurement. After that, there was a 8-week phase of running in the minimalist shoes. The runners continued their current running training routine. In the first week of this phase, participants ran in the VFF for the first 10 min, and then performed the rest of the training in their standard shoes. Each week, the time of running in minimalist shoes was extended by 5 min compared to the previous week, ending with 45 min of running in the last week. On non-running days, runners walked in the VFF for 1 h. After that phase, the fourth measurement was performed.

In this study, the Vibram Five Fingers V-run model (Vibram, Albizzate, Italy) was used. This kind of shoe is characterized by thin, flexible soles that are contoured to the shape of the human foot, including visible individual sections for the toes. It is devoid of any stabilizing or shock-absorption features^23^.

The data were collected at the research laboratory. The aim of the progressive running test was to determine heart rate (HR) and running velocity (V) corresponding to lactate threshold (LT). The test was carried out with the Insportline Club IN 557 mechanical treadmill. The conditions included a temperature of 20–23 °C and air humidity of approximately 55%. Before the test, the participants were familiarized with the testing procedure and were asked not to perform intensive physical activity in the 24 h preceding the measurement.

In our study, we have modified the running test protocol, basing on the Żołądź test for runners, which consists of 6-min runs in four heart rate zones (at, respectively, 50, 40, 30 and 20 bpm below maximal heart rate). This test is a valid and reliable method of measuring performance capacity in track and field^24^. In our study, the intensity of individual runs was modified to standardize the measurement conditions for each participant. The trial consisted of a 5-min effort separated by a 1-min interval. In the first test and measurement series, the test began with a run at a speed of 8 km/h, at a treadmill inclination angle of 1°. With each successive time interval, the speed was increased by 1.2 km/h until the lactate threshold was exceeded. The intensity of the segments was chosen to capture the moment of the lactate threshold increase, according to the definition applied by Żołądź et al.^25^. The lactate threshold was defined as the moment of increase in capillary lactate concentration by more than 0.5 mmol/L in relation to the previous interval and the increase in heart rate above 70% of the predicted maximum value^25^ calculated from the formula: HRMAX-P = 208 − 0.7 × age [in years]^24–26^. In the second, third and fourth measurements, the initial speed of the run was individually selected, taking into account the principle that the test should start at a speed of 2.4 km/h below the VLT obtained in the previous measurement. The study conducted among endurance athletes showed good to excellent repeatability (Cronbach’s alpha of 0.89–0.96) of similar protocols for lactate threshold assessment, with low intra-subject coefficient of variation. Predictive value for performance showed significant correlations ranging from 0.53–0.94^27^.

During the test, heart rate was recorded using the Polar M400 monitor (Polar Electro Oy, Professorintie 5, 90440 Kempele, Finland) with a sampling frequency of 0.2 Hz. The average value of the HR from the last 2 min of the time interval was taken into account for analyzes.

Before the test (after a 10-min rest) and immediately after each 5-min run, 5 µL of blood was collected from the fingertip for lactate concentration determination via the enzymatic-amperometric method using the Lactat EDGE analyzer (The Edge, ApexBio, Hsinchu, Taiwan). The Lactat EDGE analyzer is characterized by low measurement error and it is recommended for athlete testing^28^.

The following variables were evaluated:

- La rest—lactate concentration during rest collected from the fingertip before the test (after a 10-min rest);
- La LT—lactate concentration associated with lactate threshold;
- HR LT—heart rate associated with lactate threshold;
- V LT—running velocity associated with lactate threshold.

Statistical analysis was carried out using STATISTICA 12.0 software. The distribution of variables was assessed with the Shapiro–Wilk test. Data were presented as mean and standard deviation (SD). Two-way ANOVA with one main factor as between participants (SF Group and MS Group) and the other main factor as repeated measures was used to determine the significance of the differences regarding the evaluated variables. To assess the impact of the 8-week training program (plantar short foot exercises in SF Group or running in minimalist shoes in MS Group), measurements 1, 2 and 3 in SF Group and 1, 3 and 4 in MS Group were taken into account. Then, Tukey’s post hoc test was performed. Additionally, to check the differences between all four measurements in MS Group, one-way ANOVA was additionally conducted separately for each group. Differences were considered to be statistically significant if the level of test similarities was lower than the assumed level of significance (*p* < 0.05). Moreover, Bland–Altman plots were used to compare differences between groups and measurements. The effect size was calculated using Cohen’s d and interpreted as small (0.2–0.3), medium (0.5) or large (> 0.8)^29^.

## Results

**Figure 2:**
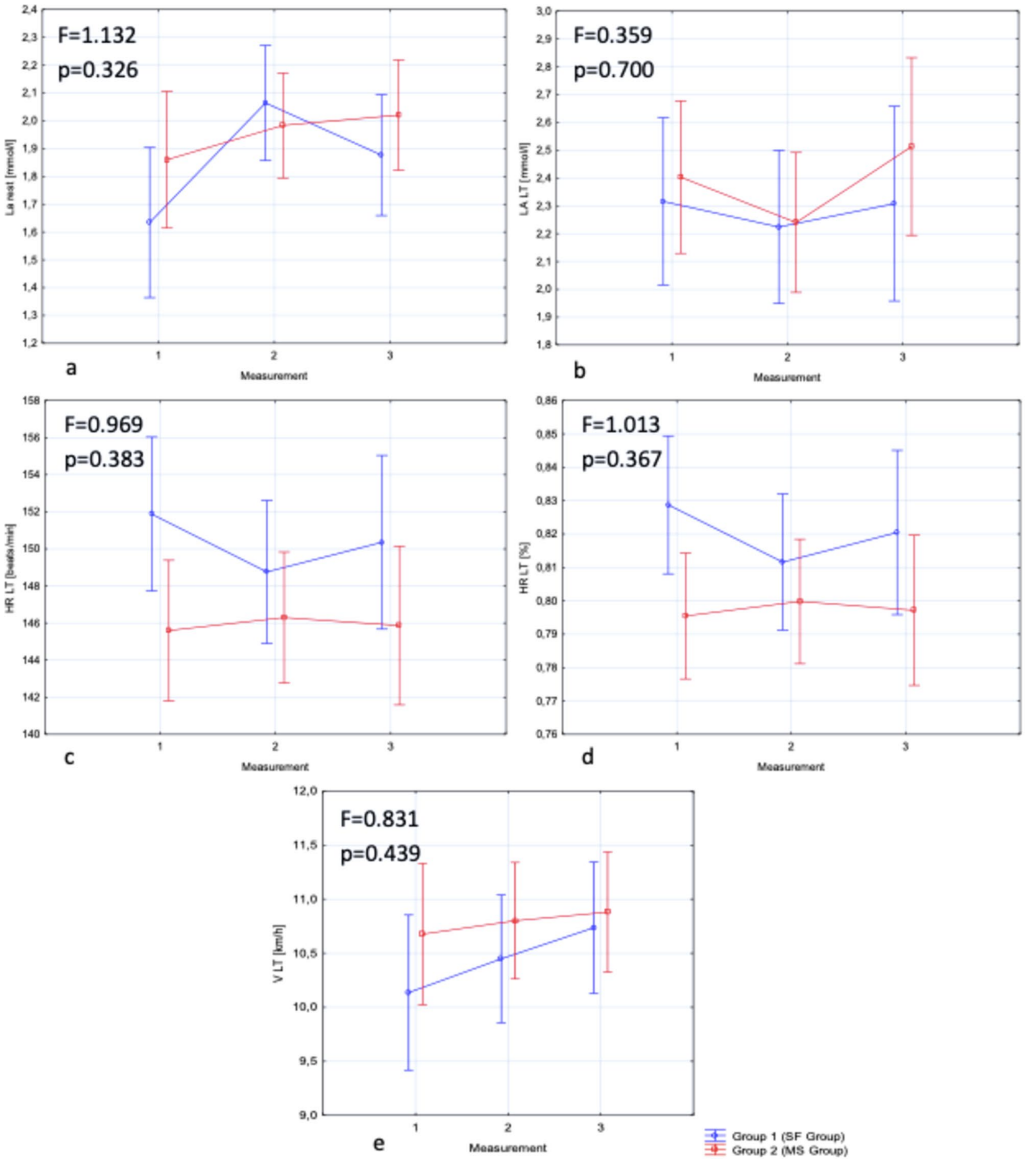
The interaction effects. (**a**) La rest [mmol/l]—lactate concentration during rest); (**b**) La LT [mmol/l]—lactate concentration associated with lactate threshold; (**c**) HR LT [beats/min]—heart rate associated with lactate threshold; (**d**) HR LT [%]—heart rate associated with lactate threshold expressed as a percentage of maximum heart rate; (**e**) V LT [km/h]—running velocity associated with lactate threshold; the vertical bars represent a 0.95 confidence interval.

After the 8-week training program, higher values of V LT were observed in both groups. This change was significant in SF Group (*p* < 0.05), where V LT increased from 10.14 to 10.74 km/h (an increase of 5.92%). In MS Group, the higher value was noted both after the stage of walking and after running in minimalistic shoes, but the changes were non-significant. In this group V LT increased from 10.68 to 10.80 km/h after the walking stage and to 10.88 km/h after the running stage (an increase of 1.12% and 1.84% relative to the baseline value, respectively). In addition, there was a significant increase in lactate concentration during rest (La rest) between the first and second measurements (*p* < 0.05) in SF Group (an increase from 1.63 to 2.06 mmol/l). After the training program, there were no significant changes in the remaining variables. In comparison, between the groups, significantly higher values of HR LT in the first measurement were observed in SF Group (*p* < 0.05). The results are presented in Table 3. The interaction effects are included in Fig. 2.

**Table 3:** Lactate concentration, heart rate and running velocity for each measurement in SF group and MS group.

	Measurement	Group 1 (SF group) (n = 25) Mean ± SD	p	ES a	Group 2 (MS group) (n = 30) Mean ± SD	p	ES a	P*	ES b
La rest [mmol/l]	1	1.63 ± 0.47	**p 1–2 = 0.001** p 1–3 = 0.090 p 2–3 = 0.231	ES 1–2 = 0.935 ES 1–3 = 0.567 ES 2–3 = − 0.418	1.86 ± 0.80	p 1–2 = 0.241 p 1–3 = 0.803 p 1–4 = 0.522 p 2–3 = 0.759 p 2–4 = 0.954 p 3–4 = 0.966	ES 1–2 = 0.344 ES 1–3 = 0.173 ES 1–4 = 0.222 ES 2–3 = − 0.221 ES 2–4 = − 0.156 ES 3–4 = 0.067	0.220	0.343
	2	2.06 ± 0.45			2.13 ± 0.77			0.566	0.109
	3	1.88 ± 0.41			1.98 ± 0.57			0.331	0.198
	4				2.02 ± 0.63				
La LT [mmol/l]	1	2.32 ± 0.76	p 1–2 = 0.819 p 1–3 = 0.999 p 2–3 = 0.847	**E**S 1–2 = − 0.115 ES 1–3 = − 0.013 ES 2–3 = 0.097	2.40 ± 0.74	p 1–2 = 0.999 p 1–3 = 0.712 p 1–4 = 0.933 p 2–3 = 0.620 p 2–4 = 0.970 p 3–4 = 0.351	ES 1–2 = 0.027 ES 1–3 = − 0.216 ES 1–4 = 0.132 ES 2–3 = − 0.240 ES 2–4 = 0.107 ES 3–4 = 0.323	0.669	0.107
	2	2.24 ± 0.62			2.42 ± 0.76			0.932	0.257
	3	2.31 ± 0.81			2.24 ± 0.74			0.389	− 0.091
	4				2.51 ± 0.92				
HR LT [beats/min]	1	151.88 ± 10.71	p 1–2 = 0.270 p 1–3 = 0.728 p 2–3 = 0.703	**E**S 1–2 = − 0.314 ES 1–3 = − 0.128 ES 2–3 = 0.143	145.60 ± 10.05	p 1–2 = 0.865 p 1–3 = 0.999 p 1–4 = 0.997 p 2–3 = 0.788 p 2–4 = 0.943 p 3–4 = 0.983	ES 1–2 = − 0.131 ES 1–3 = 0.070 ES 1–4 = 0.026 ES 2–3 = 0.201 ES 2–4 = 0.155 ES 3–4 = − 0.042	**0.029**	− 0.607
	2	148.76 ± 9.13			144.28 ± 10.09			0.350	− 0.463
	3	150.36 ± 12.98			146.30 ± 10.03			0.161	− 0.354
	4				145.87 ± 10.48				
HR-LT [%] (HR max—HR LT]	1	82.88 ± 5,49	p 1–2 = 0.270 p 1–3 = 0.731 p 2–3 = 0.699	**E**S 1–2 = − 0.348 ES 1–3 = − 0.134 ES 2–3 = 0.156	79.55 ± 4.86	p 1–2 = 0.869 p 1–3 = 0.997 p 1–4 = 0.998 p 2–3 = 0.764 p 2–4 = 0.939 p 3–4 = 0.979	ES 1–2 = − 0.154 ES 1–3 = 0.082 ES 1–4 = 0.031 ES 2–3 = 0.223 ES 2–4 = 0.175 ES 3–4 = − 0.048	**0.021**	− 0.646
	2	81.16 ± 4.34			78.80 ± 4.89			0.396	− 0.508
	3	82.05 ± 6.83			79.98 ± 5.65			0.166	− 0.333
	4				79.71 ± 5.49				
V LT [km/h]	1	10.14 ± 1.48	p 1–2 = 0.238 **p 1–3 = 0.008** p 2–3 = 0.293	**E**S 1–2 = 0.214 ES 1–3 = 0.393 ES 2–3 = 0.194	10.68 ± 2.01	p 1–2 = 0.764 p 1–3 = 0.928 p 1–4 = 0.976 p 2–3 = 0.392 p 2–4 = 0.510 p 3–4 = 0.997	ES 1–2 = − 0.059 ES 1–3 = 0.067 ES 1–4 = 0.114 ES 2–3 = 0.143 ES 2–4 = 0.196 ES 3–4 = 0.055	0.269	0.302
	2	10.45 ± 1.41			10.57 ± 1.69			0.381	0.076
	3	10.74 ± 1.57			10.80 ± 1.52			0.727	0.039
	4				10.88 ± 1.46				

Significant values are in bold.

*La rest* lactate concentration during rest, *La LT* lactate concentration associated with lactate threshold, *HR LT* heart rate associated with lactate threshold, *V LT* running velocity associated with lactate threshold, *SD* standard deviation, *p* between measurements, p* between groups, *ES a* effect size (Cohen’s d) within each group, *ES b* effect size (Cohen’s d) between study groups, *SF Group* group with short foot muscle exercises, *MS Group* group with running training in minimalist shoes.

**Figure 3:**
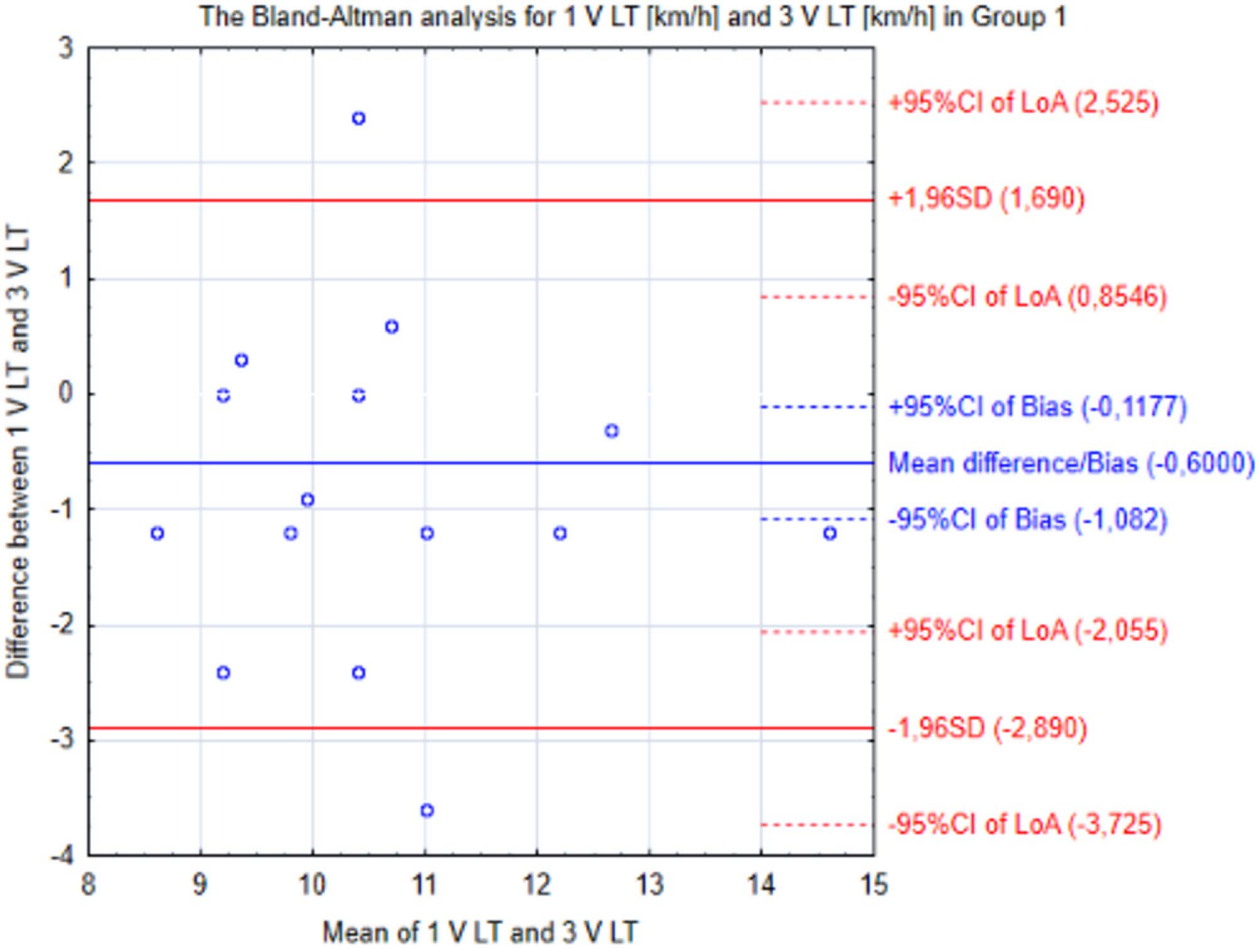
The Bland–Altman analysis for the first and last measurements in Group 1. *V LT* running velocity associated with lactate threshold, *CI* 95% confidence interval, *LoA* limits of agreement, *SD* standard deviation.

The Bland–Altman analysis showed that runners in Group 1 after training, compared to baseline, reached a 0.6 km/h higher V LT level. The mean difference (0.600) was higher than the standard error of measurement (SEM = 0.234) (Fig. 3).

**Figure 4:**
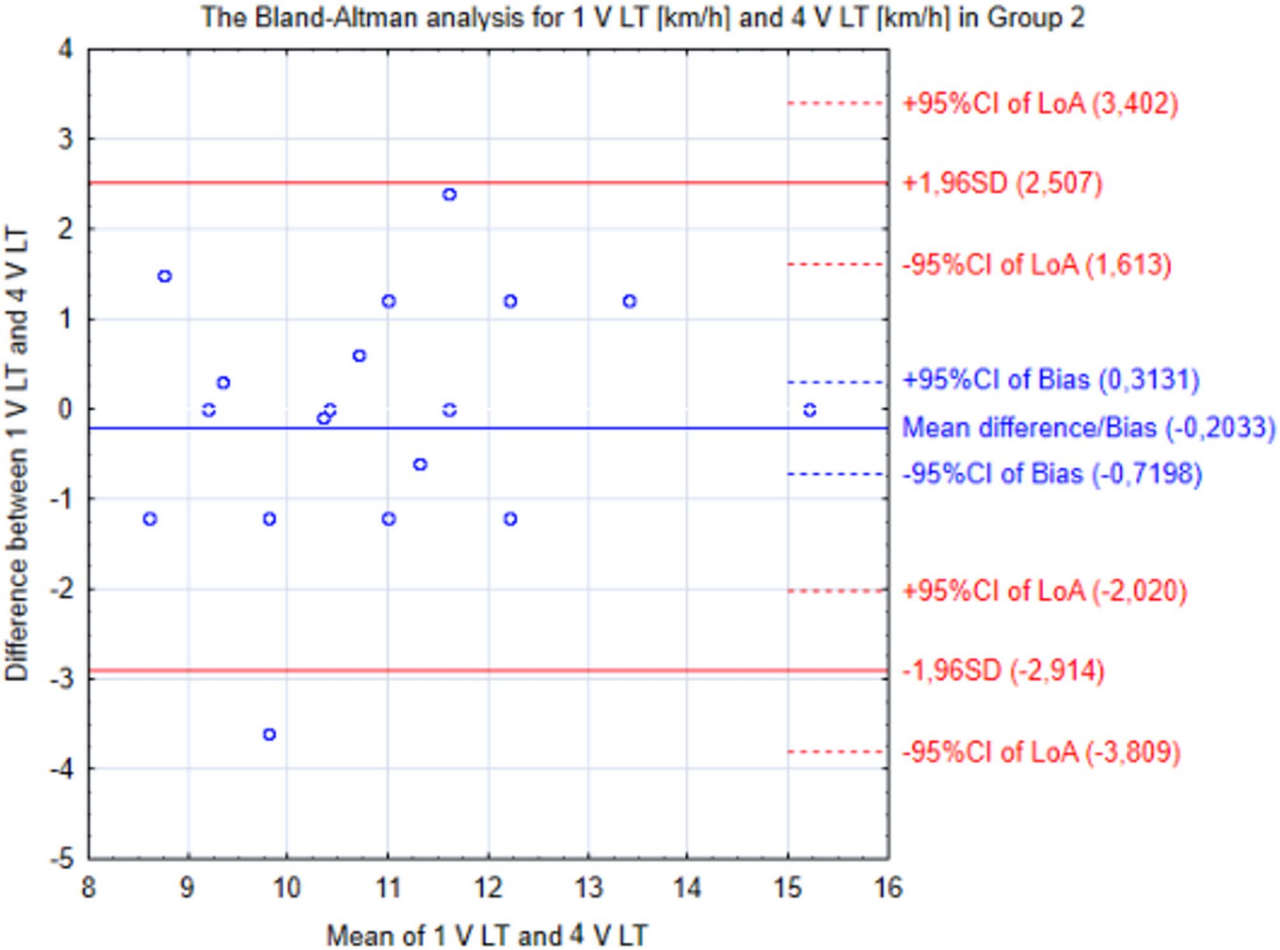
The Bland–Altman analysis for the first and last measurements in Group 2. *V LT* running velocity associated with lactate threshold, *CI* 95% confidence interval, *LoA* limits of agreement, *SD* standard deviation.

The Bland–Altman analysis showed that runners in Group 2 after training compared to baseline was 0.2 km/h higher V LT. The mean difference (0.203) was similar to the standard error of measurement (SEM = 0.253) (Fig. 4).

## Discussion

The most important observation from this study was that strengthening the foot muscles caused an increasing trend of lactate threshold velocity in the progressive running test. However, changes were significant only in SF Group, which performed direct foot muscle strengthening by exercises. Training in Vibram Five Fingers shoes caused a slightly noticeable upward trend in V LT, however, this was not statistically significant, and the magnitude of the change was close to the SEM value. Based on these results, we may suggest that direct short foot muscles exercises have a greater effect on lactate threshold velocity which is connected with running performance. Nevertheless, the training in Vibram Five Fingers shoes shows potential benefits and requires further research. Despite the importance for sports and medical training, this topic has not been sufficiently explored so far. However, there are few studies on the effect of intrinsic foot muscles strengthening through exercises or by using minimalist footwear on running economy and performance.

Modestini et al.^11^ observed significantly higher VO2 max when wearing minimalist footwear in long distance recreational runners. When compared to structured footwear, the study concludes that running in minimalist footwear demonstrates higher efficiency. Other study also indicates an improvement in running economy and respiratory exchange in minimalist shoes^12^. Squadrone et al.^30^ compared barefoot running, running in standard shoes and with the Vibram Five Fingers in male runners with a long training experience in barefoot running. VO2 was significantly lower when running in the VFF compared to standard running shoes. Other works have also indicated that efficiency in the barefoot simulated condition is greater, although the mechanical work is higher, compared to the shod condition. This was explained by enhancement of the elastic energy storage restitution at the foot arch during barefoot running and running in minimalist footwear^31–33^. The described appearance may improve the work of the foot through the use of elastic energy, while increasing the requirements for the foot muscles^15,16^. In our study, we have compared two methods of foot muscle strengthening: direct short foot muscle exercises and an indirect activation through training in minimalist footwear. An upward trend in the lactate threshold velocity after training in the VFF was observed, which may indicate positive impact of the minimalist footwear on performance in long-distance runners.

There are only a few studies in which the influence of the running training in minimalist shoes has been assessed. Warne and Warrington^34^ have measured VO2 max in the VFF and in traditional running shoes. After 4 weeks, running economy in the VFF significantly improved and became superior to shod running economy. The researchers indicated that simulated barefoot running in the VFF significantly changed running mechanics with regard to foot strike pattern towards forefoot strike pattern. Moreover, the authors suggested that the improvements may not be only related to shoe weight or design, but may have been caused by the possible influence of biomechanical and physiological adaptations induced by minimalist shoes.

Ridge et al.^35^ examined the effects of a transition from traditional to minimalist running shoes on oxygen uptake during running. After 10 weeks of training, in the group with the VFF, participants improved their running economy. The control group also showed improvement, but that change was much lower than in the experimental group. The researchers suggested that the improvement in both groups may have been the result of participating in the study and being accountable for the miles they ran. However, the average improvement in the VFF group was much higher than in the control. In our study for the first 8 weeks, participants continued their current running training, which was monitored by researchers and did not change throughout the duration of the experiment. After 8 weeks, the second measurement was performed, followed by an intervention. This allowed for the omission of running training as a confounding factor that did not change over time. In our study, we could partially confirm the results of the above research, also suggesting a positive effect of using the VFF on running performance.

Fuller et al.^13^ demonstrated that training in minimalist shoes caused small improvement in the 5-km time-trial performance and moderate improvement in running economy compared to training in conventional shoes. The results of our work seem to be in line with the above results. We have observed an upward trend in lactate threshold velocity after training in the VFF, which may indicate a positive effect of the minimalist footwear on the performance in long-distance runners. However, despite the observed increasing trend, this result was not statistically significant.

Researchers have long suggested that an important aspect in human running is the longitudinal arch of the foot. The plantar arch absorbs impact forces and helps to maintain midtarsal rigidity for powered plantar flexion during toe-off. During running, the elastic structures of the plantar arch work as a spring, returning approximately 17% of the energy generated during each stance phase^36^. A crucial role in supporting the medial longitudinal arch, providing the foot stability and flexibility for shock absorption, is played by the plantar intrinsic foot muscles^37^. There are few studies regarding the effects of using minimalist footwear on the short foot muscles. Curtis et al.^17^ observed that wearing minimal shoes for six months, even for non-intensive daily activities, increases toe flexion strength. Campitelli et al.^15^ indicated that wearing the VFF significantly increased intrinsic muscle thickness of the abductor hallucis measured using ultrasound at baseline, after 12 weeks, and following 24 weeks of the training program. The authors suggested that the observed appearance, with presumed associated strengthening of that muscle, may support the medial longitudinal arch, which may subsequently better control pronation and result in less pronatory foot injuries.

Similarly, Johnson et al.^16^ showed that the cross-sectional area of the abductor hallucis muscle significantly increased in the VFF group during the 10-week training protocol compared to the traditional shoe group. The authors suggest that the observed changes would theoretically correspond to an increase in strength of the abductor hallucis, although strength was not directly measured. Based on the results from the cited studies, it can therefore be assumed that running in minimalist footwear can strengthen the short foot muscles. In our study, we have compared direct short foot muscle exercises and indirect activation through training in the Vibram Five Fingers. Beneficial changes were seen in both groups, but direct short foot muscle activation through exercises was more effective, causing statistically significant changes.

Although the influence of intrinsic foot muscle strengthening has been discussed, there are very few studies on the impact of this kind of exercises on running economy and performance. Goldman et al.^9^ reported the effectiveness of 7-week strength training of the toe flexor muscles and athletic performance. After the training period, significant improvement in horizontal jump distance was observed with no changes in vertical jump. Probably, after strength training, the toe flexor muscles depressed the toes against the ground more effectively; therefore, the jumpers were able to lean forward more and flatten their take-off angle during the horizontal jump. The researchers suggested that the toe flexor muscles may contribute to force generation during a lean-forward type of movement. This can be used during running. The results obtained in our study indicated that the short foot muscle exercises were effective concerning improvement of lactate threshold velocity in the progressive running test.

Hashimoto and Sakuraba^10^ observed significantly higher values of foot flexor muscle strength and improvement in vertical jump, the one-leg long jump and 50-m run time tests after 8-week strengthening of the foot flexor muscles. The authors suggested that improvement of all dynamic tests resulted from increased foot hardness that was accompanied by improved intrinsic foot flexor strength and arch formation. In our study, we have also observed improvement in running performance, manifested as an increase in lactate threshold velocity.

In the research carried out by Sulowska et al.^14^, it was indicated that a 6-week short foot muscle exercises program improved results in the Running-Based Anaerobic Sprint Test (RAST). The authors concluded that the exercises of plantar short foot muscles may have beneficial effects on the performance of the lower extremity muscles by improving energy transfer through body segments and increasing values of generated power. The authors further indicated a multifactorial relationship between the foot and the upper segments of the body, including both the skeletal system—the impact of foot pronation on proximal segments of the kinematic chain and interconnections within myofascial structures. According to the Anatomy Trains paradigm, the foot is a component of four main anatomy trains: the superficial back line, the superficial front line, the lateral line and the spiral line, which participate in the transfer of energy^38^. The results of our current study seem to confirm these assumptions. Strengthening the short foot muscles improved running performance. Based on previous research, we can assume that this improvement may be related to enhancement of elastic energy storage restitution at the foot arch.

There are some limitations to our study that should be addressed. First of all, the total running distance of the participants was between 20 and 100 km per week, which may have influenced the group homogeneity. Certainly, it would be worth assessing running economy with oxygen uptake measurements. In our study, we based on the definition of the lactate threshold provided by Żołądź et al.^25^. However, the running test protocol was modified, which can be considered a limitation of this study and may affect the interpretation of this trial given the lack of validity of the protocol adopted by the authors. Moreover, it would be wise to carry out a progressive running test in the Vibram Five Fingers for participants who have trained in the minimalist shoes. The next limitation is that we have not assessed the foot strike pattern. Another factor may be that the runners in the group using the Vibram Five Fingers performed only part of their training in the minimalist shoes. In the running stage, runners began with 10 min of running in the minimalist shoes in the first week, increasing the running time by 5 min each week, ending with 45 min. Perhaps if the research was continued and runners performed longer training in minimalist shoes, then, the effect in this group could be greater. It would be worth expanding the research and assessing the impact of using the Vibram Five Fingers during whole running training unit. Further research is recommended.

## Conclusion

The results obtained in our study allow to suggest that strengthening of the short foot muscles may improve lactate threshold velocity which is connected with running performance in long-distance runners. Direct short foot muscles exercises have a greater effect on running performance. Nevertheless, the training in Vibram Five Fingers shoes shows potential benefits and requires further research. Our research was the first to compare these two kinds of training programs, engaging the short foot muscles and evaluating their effect on the lactate threshold velocity. The observed changes may be induced by an increase in elastic energy storage restitution following foot interventions. Moreover, the strengthening of the short foot muscles may improve energy transfer through body segments resulting in beneficial effects on the body performance. Considering the positive impact of strengthening the short foot muscles on running performance, it is worth reflecting on the implementation of these methods in the training of long-distance runners. Further research is necessary to investigate other aspects, including the impact of short foot exercises on performance in sports competitions and the occurrence of injuries.

## Data Availability

All data generated or analyzed during this study are included in this published article.
